# A Patient with MSUD: Acute Management with Sodium Phenylacetate/Sodium Benzoate and Sodium Phenylbutyrate

**DOI:** 10.1155/2017/1045031

**Published:** 2017-05-15

**Authors:** Melis Köse, Ebru Canda, Mehtap Kagnici, Sema Kalkan Uçar, Mahmut Çoker

**Affiliations:** ^1^Pediatric Metabolism Department, Behçet Uz Children's Training and Research Hospital, Izmir, Turkey; ^2^Pediatric Metabolism Department, Ege University Medical Faculty, Izmir, Turkey

## Abstract

In treatment of metabolic imbalances caused by maple syrup urine disease (MSUD), peritoneal dialysis, and hemofiltration, pharmacological treatments for elimination of toxic metabolites can be used in addition to basic dietary modifications. Therapy with sodium phenylacetate/benzoate or sodium phenylbutyrate (NaPB) in urea-cycle disorder cases has been associated with a reduction in branched-chain amino acid (BCAA) concentrations when the patients are on adequate dietary protein intake. Moreover, NaPB in treatment of MSUD patients is also associated with reduction of BCAA levels in a limited number of cases. However, there are not enough studies in the literature about application and efficacy of this treatment. Our case report sets an example of an alternative treatment's efficacy when extracorporeal procedures are not available due to technical difficulties during attack period of the disease.

## 1. Introduction

Maple syrup urine disease (MSUD) is an autosomal recessive inherited metabolic disease caused by decreased enzyme activity of branched-chain alpha-ketoacid dehydrogenase complex (BCKDC), responsible for catabolism of leucine (leu), valine (val), and isoleucine (isoleu). Global known incidence rate of this condition is 1 in 185000 [1]. BCKCD is a 4-million Dalton macromolecule and consists of heterodimeric E1 decarboxylase component (E1a and E1b subunits), E2 transacylase that consists of 24 subunits and 1 homodimer E3 component [2]. The condition shows in five different clinical phenotypes as classic, intermediate, intermittent, thiamine-responsive and E3 deficiency type [3].

Formerly, studies have shown that a drop in plasma branched-chain amino acid levels in cases with urea-cycle metabolism disorders can be obtained with sodium phenylacetate/benzoate or sodium phenylbutyrate (NaPB) treatments [4, 5]. Following the path of these studies, new studies about using nitrogen scavenging agents in MSUD patients to reduce branched-chain amino acid levels are also in progress [6]. However, as with all branched-chain amino acid metabolism disorders, extracorporeal methods are still the first line of choice in treatment of encephalopathy caused by acute decompensation [7].

In this article, our main aim is to share our experience in a MSUD patient under hemodialysis for acute metabolic decompensation using Na phenylacetate/benzoate followed by NaPB to reduce the increased branched-chain amino acid levels after stopping hemodialysis for technical reasons.

## 2. Case

A 2-day-old male infant was hospitalized in pediatric intensive care unit with encephalopathy findings of repetitive myoclonic convulsions, hypertonicity, and pedaling. The parents of the infant were first-degree cousins. The patient had deep metabolic acidosis and his plasma amino acid levels were leu: 5933 *µ*mol/l (*N*: 33–124), isoleu: 1440 *µ*mol/l (*N*: 27–110) (NR for appropriate age), val: 1345 *µ*mol/l (*N*: 30–125), and urine organic acid result was 3-Ketoisocaproic acid 1328 mmol/molCrea. A genetic analysis for MSUD was performed following these results and a homozygote mutation on DNT gene IVS7-1G>A was found. Leu levels of the patient were reduced to normal ranges after 72 hours of successful hemodialysis. Moreover, ketoacid exertion through urine was also significantly reduced following hemodialysis. Patient was treated with isoleu, leu, and val-free formula and 10 mg/day thiamine. Despite seeing no complete elimination in urine ketoacid during 4 weeks of thiamine treatment and blood leucine levels showing a wavy pattern against dietary modifications, thiamine treatment was increased up to 200 mg/day step by step. However, patient showed no response to thiamine treatment. Following initial decompensation attack, medical supportive treatments were used during increases in blood leucine, valine, and isoleucine levels during infections for 19 months' follow-up period. Patient had normal development during follow-up period and his weight was 75–90 p, height was 90–97 p, circumference of head was in 25–30 percentile, unassisted sitting was possible and speaking 1-2 words in 19/12 months.

**Figure 1:**
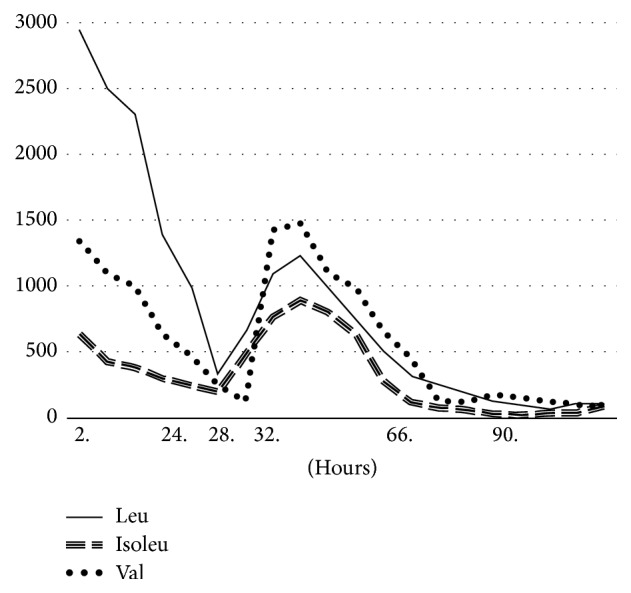
Hemodialysis and Na phenylacetate/benzoate and phenylbutyrate treatment during attack management. 0–28 hrs: Continious venovenous dialysis (through femoral vein) 28. hr: Cloggling of hemodialysis catheter, 32–66. hrs: Na phenylacetate/benzoate, 66. hrs+: Na phenylbutyrate (Pheburane® 483 mg/gr, granulated).

On 19 months of age patient was taken to emergency department for emesis, reduction in oral intake, and sleepiness. His physical examination during initial admission showed moderate dehydration and lethargy. Laboratory analyses were consistent with metabolic acidosis and hyperammonemia (which showed that pH: 7.,21, HCO_3_^−^: 16 mmol/l, and NH_3_: 212 mmol/l). He has been treated by high-dextrose fluid and intralipids (glucose: 14.5 mcg/kg/min, lipid: 3 gr/kg/day, and l-carnitine 100 mg/kg/day) following an initial intravenous fluid bolus. In addition, patient received continuous nasogastric infusion of BCAA-free formula as well as isoleucine and valine supplements to promote leucine incorporation into newly synthesized proteins. Over the last 6 hours, patient's neurological status deteriorated progressively with decreased levels of consciousness and decreased motor reflexes. CT scans showed no cerebral edemas within brain. Hemodialysis was ordered after seeing the patient's blood amino acid levels (leu: 2948 *µ*mol/l (*N*: 33–124), isoleu: 438 *µ*mol/l (*N*: 27–110), and val: 1345 *µ*mol/l (*N*: 30–125)) with the patient in encephalopathy state. The patient has been transferred to intensive care unit and connected to a ventilator. Eight Fr double-lumen hemodialysis catheter was placed into femoral vein and continuous venovenous hemodialysis (CVVHD) was started. Patient's blood amino acid levels and urine organic acid levels were analyzed every 2 hours. Leucine levels were reduced to 328 *µ*mol/l after 24 hours of hemodialysis. On 28th hour of hemodialysis, patient had a fever and he had infection criteria but infection focus could not be detected. Hemodialysis was paused after this infection situation and simultaneous clogging of femoral catheter. Blood leucine level of patient rose to 1100 *µ*mol/l on 4th hour of stopping hemodialysis. Status epilepticus was seen in patient with generalized tonic-clonic convulsions. Bedside electroencephalogram showed generalized epileptic activity and control brain CT showed no brain edema. Midazolam and levetiracetam infusions were started. Since extracorporeal elimination treatments cannot be performed at this stage, patient was loaded with Na phenylacetate/Na benzoate (Ammonul %10/%10 50 ml), with a dosage of 250 mg/kg for 2 hours, and then 250 mg/kg/24-hour infusion, based on previous studies on reducing branched-chain amino acid levels in urea-cycle disorder patients using Na phenylacetate/Na benzoate and NaPB [4–6]. Blood leu-isoleu and val levels were checked every 2 hours and urine organic acid was checked every 4 hours. Patient regained full consciousness following 24-hour infusion of Na phenylacetate/Na Benzoate infusion and his blood leucine level was 521 *µ*mol/l. Na phenylacetate/Na benzoate treatment was stopped at the 24th hour. NaPB was started enterally through nasogastric tube with 500 mg/kg/day dosage after Na phenylacetate/Na benzoate had been stopped in order to continue leu levels constantly in normal ranges. On 36th hour blood leucine level was 321 *µ*mol/l and patient was separated from the mechanical ventilator. After 12 hours of follow-up, Patient's BCAA and BCKA levels were shown on Figure 1. Patient was discharged following NaPB treatment and BCAA-restricted dietary modifications on 17th day of admission. Pheburane (483 mg/gr, granulated) was chosen since it was taste-masked and odor-free with a dosage of 500 mg/kg/day. His branched-chain amino acid levels were kept down within normal values under NaPB treatment during hospitalization. During discharge of the patient, neurological examination showed spasticity of lower extremity so baclofen treatment was started. In 1-year follow-up, branched-chain amino acid levels of the patient were within normal range (Table 1). Natural protein intake of the patient was increased in his diet under NaPB treatment and his dependency on the diet was reduced (Table 2). No attacks were seen. Spasticity completely improved and patient had ordered walking and speech with 9-10 words in 2 years and 5 months of age. After one year follow-up his weight was 50–75 p, height was 90 p, circumference of head was in 25 percentile.

## 3. Discussion

Sodium phenylacetate/sodium benzoate and NaPB are alternative pathways for excretion of waste nitrogen in urea-cycle disorders using endogenous biosynthetic pathways to eliminate nonurea waste nitrogen as a substitute for defective urea synthesis. They work by stimulating nitrogen excretion as phenylacetylglutamine and hippuric acid. Phenylbutyrate does not accumulate in plasma and converted into phenylacetate which is its active form. It is then conjugated with glutamine in liver and kidney to form phenylacetylglutamine which is excreted in urine, replacing urea as a mean of eliminating excess nitrogen compounds [4, 5, 8].

One of the hypotheses that explain the reduction of BCAA levels using NaPB and Na phenylacetate is that overproduction of phenylacetylglutamine depletes glutamine reserves and secondarily reduces BCAA levels [5]. In vitro and animal studies showed that active metabolite of NaPB increased BCKDC complex activity by inhibiting phosphorylation of E1a subunit and its inactivation, which is responsible for breakdown of BCAA [6, 9]. In a 5-person group of both classic and late-onset MSUD cases, patients were given NaPB for treatment and 3 out of 5 showed a significant decrease in branched-chain amino acid levels independent of residual BCKDH activity [6]. This response sparked interest in using NaPB as a recent approach in treatment of MSUD, which has no pharmacological treatment method apart from thiamine-responsive form. A randomized double blind placebo controlled study is underway to investigate whether NaPB may be an effective therapy for patients with MSUD (ClinicalTrials.gov Identifier: NCT01529060).

The genetic analysis of our patient revealedIVS7-IG>A mutation in DBT gene. DBT gene is responsible for coding E2 subunit of BCKDC and its molecular phenotype is compatible with Type II MSUD [1]. As known, there is a strong correlation between thiamine-responsive MSUD with mutant E2 protein [3] but since our patient did not respond to a gradual thiamine treatment from 10 mg/day to 200 mg/day, he was deemed as thiamine-unresponsive [10]. Patient's BCKDC enzyme levels could not be measured; however, his clinical presentation showed that his clinical phenotype is compatible with classic MSUD [1, 3]. Brunetti-pierri et al. [6] showed that NaPB's effect is mainly on E1a subunit of BCKDC; however the same study showed a significantly reduced level in BCAA levels following NaPB treatment in both patients with E2 mutations. However, up to now, there is no data about using sodium phenylacetate/sodium benzoate and NaPB effectively in a MSUD patient during an acute decompensation attack. In our patient, a speedy and efficient drop in BCAA levels was obtained using sodium phenylacetate/sodium benzoate during attack phase and hemodialysis had to be stopped due to technical reasons. In addition, NaPB treatment was continued after the attack and patient's dependency to diet was reduced to an extent and natural protein intake was increased after 1-year of follow-up (Table 2).

## 4. Conclusion

It is a known fact that sodium phenylbutyrate usage increases enzyme activity of alpha-ketoacid dehydrogenase, effectively decreasing blood branched-chain amino acid levels. However, there is still not enough data on the usage and efficacy of sodium phenylbutyrate during MSUD attack periods. The case we discussed here in our paper could be possibly interpreted as the first example of an alternative attack management method in situations where extracorporeal procedures are not available due to technical reasons.

## Figures and Tables

**Table 1:** Values of plasma leucine, isoleucine, valine, alanine, glutamine, glycine, and ammonia during periods of therapy.

	Normal values	0–19 months	NaPB 17th day (discharging)	NaPB 3 months	NaPB 6 months	NaPB 12 months
Leucine (*µ*mol/L)	33–124	422 ± 25,8	108	142	167	138
Isoleucine (*µ*mol/L)	27–110	78 ± 34,2	92	87	52	67
Valine (*µ*mol/L)	30–125	176 ± 28,1	98	101	119	121
Alanine (*µ*mol/L)	54–250	34 ± 54,7	234	207	267	283
Glutamine (*µ*mol/L)	80–450	198 ± 49,5	267	199	301	276
Glycine (*µ*mol/L)	50–230	436 ± 38,1	182	178	167	176
Ammonia (*µ*mol/L)	<70	111 ± 28,1	101	77	63	92

**Table 2:** Nutritional treatment plan during periods of therapy.

	After diagnosis	19 mo of age (before attack)	Acute management 12–72 hours	Acute Management After 72 hours	NaPB 17th day (Discharging)	NaPB 3 months	NaPB 6 months	NaPB 12 months
Leu (mg/day)	336	360	0	360	360	390	420	450
Isoleu (mg/day)	161	298	300	290	290	342	390	410
Valine (mg/day)	158	287	300	280	280	300	320	330
Protein (gr/kg/day)	2,7	2,5	2,5	2,9	2,95	2,8	2,85	2,92
Energy (Kcal/day)	110 kcal/kg/day	1198	1400	1300	1450	1580	1620	1700
